# Has the human biological interaction with SARS‐CoV2 variants entered a pliant “Faustian bargain”?

**DOI:** 10.1002/prp2.1244

**Published:** 2024-07-09

**Authors:** Richard J. Head, Marko Popovic, Jennifer H. Martin

**Affiliations:** ^1^ Drug Discovery and Development, Clinical and Health Sciences University of South Australia Adelaide South Australia Australia; ^2^ Institute of Chemistry, Technology and Metallurgy University of Belgrade Belgrade Serbia; ^3^ Centre for Drug Repurposing and Medicines Research University of Newcastle and Hunter Medical Research Institute Newcastle New South Wales Australia

**Keywords:** COVID‐19, Gibbs free energy, thermodynamics

## Abstract

We hypothesize that a “Faustian bargain”—the trading of increased SARS—CoV2 viral infection with a concurrent potential for prevention of life‐threatening lower lung infection explains the previous and future morbidity and mortality from COVID‐19. Further, this trade‐off is made feasible by fundamental principles of thermodynamics and receptor affinity.

We have highlighted recently^1^ the importance of the affinity of SARS‐CoV2 for its target the Angtiontensin II (ACEII) receptor, and in fundamental thermodynamic terms, this affinity is linked to the passage of the virus from the airway and its subsequent diffusion across the mucosa. This relationship of infectivity and pathophysiology in the upper airway is key to understanding the morbidity and mortality of this pandemic. For example, the dissociation constant, Kd, of SARS‐CoV is 5.0 nM, while that of SARS‐CoV‐2 is 1.2 nM, at 30°C.^2^ These values can be used to find Gibbs energy of binding, which is: Δ_*B*_ *G* ^*0*^ = −48.2 kJ/mol for SARS‐CoV and Δ_*B*_ *G* ^*0*^ = −51.8 kJ/mol for SARS‐CoV‐2. Thus, SARS‐CoV‐2 has a 1.075 × greater binding affinity than SARS‐CoV.

With a high affinity of SARS‐CoV2 for the ACEII receptor in the nasopharyngeal region, the virus will be drawn from the upper airway, leading to a relatively survivable upper airway respiratory infection. In contrast with lower affinity SARS‐CoV2 mutants, there will be poorer upper airway viral extraction and a higher risk of viral migration to the lower lung, with potentially life‐threatening consequences, as seen early on in the pandemic. Thus, as the affinity increases with SARS‐CoV2 variants, the resultant upper airway infection favors both viral spread in the population as well as engendering a greater potential for population survival. For completeness, the binding affinities for the SARS‐CoV‐2 variants can be found in [4].

There is thus a convergent and common opportunity for the virus and for human, that is, a modified “Faustian bargain” based upon a convergent and shared advantage.^3^ Indeed, an analysis of the appearance of mutations through time evolution of SARS‐CoV‐2 from Hu‐1 to the latest XBB.1.9.1 and XBB.1.16 variants shows a decrease in Gibbs energy of binding, equivalent to increase in affinity, increasing antigen–receptor binding, virus passage through the mucosa, and increased infectivity.^4^, ^5^, ^6^

The “Faustian bargain” is thus the trading of the possibility of increased viral infection in the population with a concurrent potential for prevention of life‐threatening lower lung infection made feasible by the fundamental principles of thermodynamics.

## AUTHOR CONTRIBUTIONS

The original idea is from RIchard Head. All authors contributed to the development of the hypothesis, analysis and writing of the article.

## ETHICS STATEMENT

Not applicable.

## Data Availability

Not applicable.
